# Use of Landsat Land Surface Temperature and Vegetation Indices for Monitoring Drought in the Salt Lake Basin Area, Turkey

**DOI:** 10.1155/2014/142939

**Published:** 2014-01-21

**Authors:** Osman Orhan, Semih Ekercin, Filiz Dadaser-Celik

**Affiliations:** ^1^Salt Lake Water and Environmental Research Center, Aksaray University, 68100 Aksaray, Turkey; ^2^Department of Geomatics Engineering, Faculty of Engineering, Aksaray University, 68100 Aksaray, Turkey; ^3^Department of Environmental Engineering, Faculty of Engineering, Erciyes University, Kayseri, Turkey

## Abstract

The main purpose of this paper is to investigate multitemporal land surface temperature (LST) changes by using satellite remote sensing data. The study included a real-time field work performed during the overpass of Landsat-5 satellite on 21/08/2011 over Salt Lake, Turkey. Normalized vegetation index (NDVI), vegetation condition index (VCI), and temperature vegetation index (TVX) were used for evaluating drought impact over the region between 1984 and 2011. In the image processing step, geometric and radiometric correction procedures were conducted to make satellite remote sensing data comparable with *in situ* measurements carried out using thermal infrared thermometer supported by hand-held GPS. The results showed that real-time ground and satellite remote sensing data were in good agreement with correlation coefficient (*R* ^2^) values of 0.90. The remotely sensed and treated satellite images and resulting thematic indices maps showed that dramatic land surface temperature changes occurred (about 2°C) in the Salt Lake Basin area during the 28-year period (1984–2011). Analysis of air temperature data also showed increases at a rate of 1.5–2°C during the same period. Intensification of irrigated agriculture particularly in the southern basin was also detected. The use of water supplies, especially groundwater, should be controlled considering particularly summer drought impacts on the basin.

## 1. Introduction

Remote sensing is extremely useful for understanding the spatiotemporal land cover change in relation to the basic physical properties in terms of the surface radiance and emissivity data. Since the 1970s, satellite-derived (such as Landsat Thematic Mapper-TM) surface temperature data have been utilized for regional climate analyses on different scale [1–3].

A drought index, which describes the temporal and spatial variations of crop water use status, can be suitable for drought monitoring. As climatic data, such as precipitation and air temperature, collected by weather stations have poor spatial resolution, satellite remotely sensed data offer considerable advantages and should be an integral part of monitoring drought, especially for detecting the temporal and spatial evolution of drought. Landsat series of satellites have been used to generate vegetation indices such as the normalized difference vegetation index (NDVI) and to retrieve land surface temperature (LST). NDVI not only maps the presence of vegetation on a pixel basis, but also provides measures of the amount or condition of vegetation within a pixel. LST is a good indicator of the energy balance at the Earth's surface because it is one of the key parameters in the physics of land-surface processes on regional and global scales.

Identification of change over a period at regional level is one of the main requirements to analyze the climate change. LST is one of the most important environmental parameters used in determining the exchange of energy and matter between the surface of the earth and the lower layer of the atmosphere. Continuous monitoring of this parameter is likely to yield information about the suspected climate change [4].

NDVI, vegetation condition index (VCI), and temperature vegetation index (TVX) have been widely used for determining temporal LST changes and monitoring drought [1–15]. Falahatkar et al. [10] used the technique of image differencing to produce a radiant temperature change image using the normalization of the surface radiant temperature to understand the impacts of land cover change on surface radiant temperature. The temporal study by Rajasekar and Weng [11] used contour to visualize the change in the concentration of heat with respect to time.

Lambin and Ehrlich [14] used VCI to estimate vegetation health and monitor drought. Among land-use classes, the maximum VCI value of 92.1% was observed in onions for the excess year, whereas groundnut witnessed the maximum values of 78.2, 64.5, and 55.2% for normal, deficit, and drought years, respectively. TVX provided a simple way to combine remotely sensed thermal and reflected radiation by calculating their ratio.

TVX is negatively related to water conditions. The major advantage of TVX is that it integrates both the reflective and thermal bands of remotely sensed data, which offers more spectral information for drought detection [13].

This study aims to monitor drought at Salt Lake and its basin area located in Central Anatolia, Turkey, using multitemporal Landsat-5 TM imagery.

## 2. Methodology

### 2.1. Study Region

**Figure 1:**
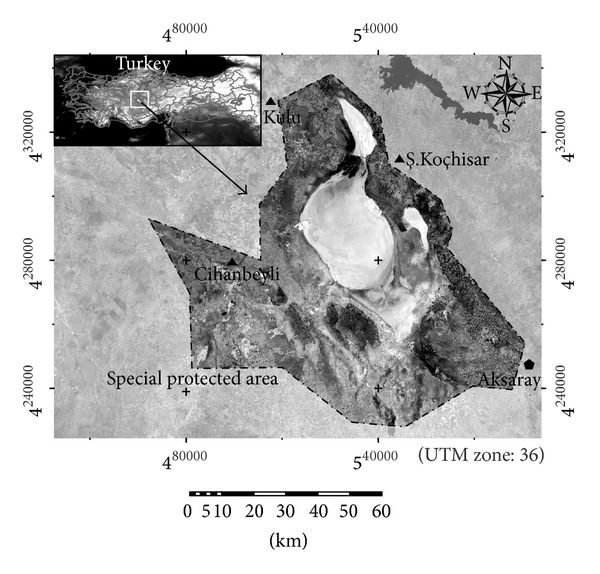
Location of the Salt Lake Basin (Turkey) through Landsat-5 TM near infrared band (August 2011).

The Salt Lake is a saline lake occupying a huge area in the arid central plateau of Turkey, about 65 miles (105 km) northeast of Konya, neighbouring also Nigde and Ankara provinces (Figure 1). It is the second largest lake of Turkey, after Lake Van, lying at an elevation of 2,970 feet (905 m) as a tectonic lake. Despite its huge area (580 sq miles or 1500 sq kilometres), for most of the year, it is very shallow (between 0.5 and 1 meters), especially during dry summer months when water evaporates in huge quantities leaving a tick crust of salt on the surface up to 30 centimetres. This salt is extracted, worked, refined, and sold in the local market, thus making this the biggest industry for small towns' economy in the area. It is the largest salt lake of Turkey. The density of the water is 1.225 gr/cm^3^ and salt content is 32.4%. The lake has no outlet, and only few surface streams feed it but they dry in summer when the weather is hot. Precipitation in the surrounding area is as low as 10 inches (250 mm) per year (AAT, 2007).

### 2.2. *In Situ* Measurements

One of the major problems in the validation of remote sensing data with ground truth observation is the dissimilarity between the spatial scales of field thermometers (<1 m^2^) and that of satellite sensors (120 by 120 m^2^ for Landsat-5 thermal infrared-TIR). The comparison of ground (point) measurement with that of satellite (area averaged) data is meaningful only when the test site is homogeneous in both temperature and emissivity at various spatial scales involved. Accuracy of ground measurements must be assessed, including the natural variability of the surface. Ideal validation of a test site is very difficult to achieve; however, crusted saline soil, bare saline soil, salt, and fallow area can be used as most suitable sites for validation [16].

**Figure 2:**
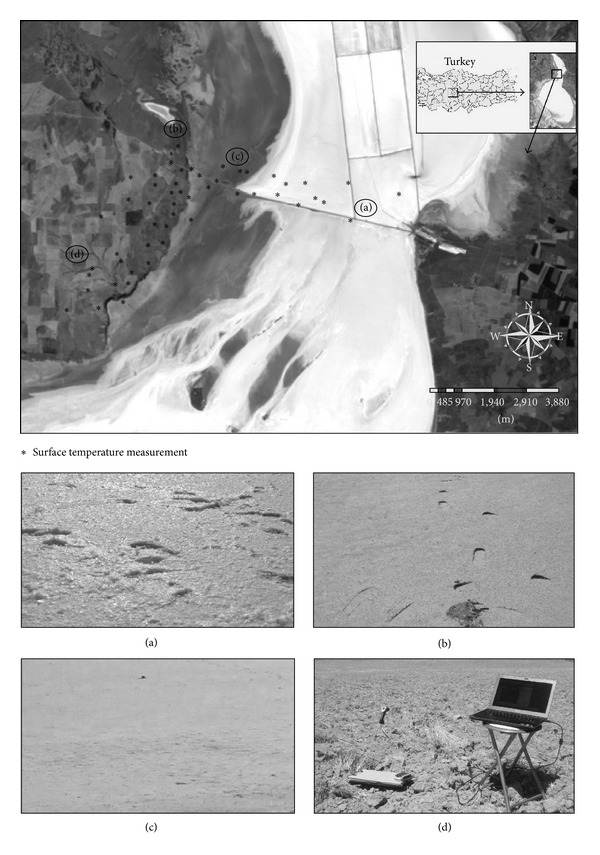
The details of the real-time field measurements performed with the overpass of Landsat-5 (real-time measurements were collected on August 21, 2011 07:30–11:00 AM, GMT+2). Landsat-5 overpass: 08:21 AM over the Salt Lake, Turkey (path/row = 177/033). (a) Salt, (b) bare saline soil, (c) crusted saline soil, and (d) fallow area.

The real-time surface temperature measurements were carried out on August 21, 2011 (07:30–11:00 AM, GMT+2) over crusted saline soil, bare saline soil, salt, and fallow area during daytime, cloud-free concurrently to the overpasses time of Landsat-5 at 08:21 AM (GMT+2) over the Salt Lake, Turkey (Path/Row = 177/033). 42560 Model Infrared Thermometer with Wireless PC Interface was used for collecting ground surface temperatures. 42560 Model of 0.1°C resolution measures ground temperature in single channel (8–14 *μ*m) with an accuracy of ±0.5°C. In order to capture the spatial variability of the surface temperature within the land-use class at measured site, several readings were recorded at intervals of about 100 m around the site (Figure 2). Average ground temperatures over the land-use classes at various measured sites were estimated with maximum standard deviation of <0.5°C for all locations [17].

### 2.3. Satellite Remote Sensing Data

Landsat-5 multispectral image series were used as remote sensing data source for the study (Table 1). A cloud-free Landsat-5 TM image (path/row = 177/033) acquired on August 21, 2011 (08:21, GMT+2) was used as real-time satellite remote sensing data.

In the image processing step, satellite remote sensing data were geometrically transformed to real-world coordinates using UTM projection and WGS 84 datum. The SPOT-5 Pan data having 5 meter spatial resolution (acquired on April 14, 2005) and base maps (1 : 25 000) were used for the ground control, resulting in a root mean square (RMS) accuracy of less than a half pixel utilizing approximately 50 ground control points for each image. Table 1 presents the details of the geometric correction process. The nearest neighbour resampling method [18, 19] and a second-order polynomial transformation method [20] were used to create the output images with 30 m ground resolution for Landsat-5 sensor data. Image processing procedure and the evaluation of the multitemporal satellite remote sensing data were performed using ERDAS Imagine and ArcGIS software packages.

### 2.4. Data Processing

**Figure 3:**
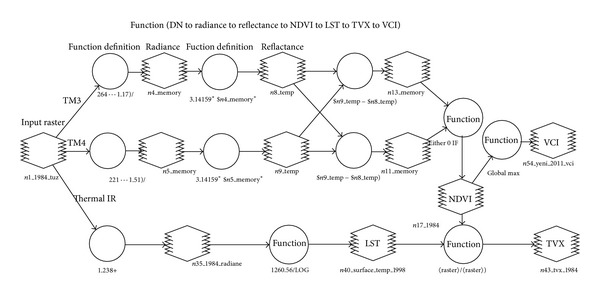
Presentation of flow chart used for producing vegetation index by using Modeler algorithm of ERDAS Imagine image processing software.

Figure 3 provides general overview about the details of procedure carried out for producing LST and vegetation index maps by using Modeler algorithm of ERDAS Imagine image processing software.

#### 2.4.1. LST

Landsat-5 TM images for the month of August (1984, 1989, 1998, 2003, 2007, and 2011) were used for generating LST maps. First, Landsat-5 TM, 2011, and then other historical images were radiometrically corrected to be able to compare with *in situ* infrared thermometer measurements and with each other [21]. The aim of radiometric correction is to convert remotely sensed digital numbers (DN) to LST values in order to make the data comparable. Equation (1) is used to perform conversion from digital number (DN) to spectral radiance (*L*):

(1) $$\begin{array}{c} L_{\lambda }=L_{\min}+\left(L_{\max}-L_{\min}\right)*\frac{\text{DN}}{255}, \end{array}$$

where

- *L* = Spectral radiance,
- *L*_min⁡_ = 1.238 (Spectral radiance of DN value 1),
- *L*_max⁡_ = 15.600 (Spectral radiance of DN value 255),
- DN = Digital Number.

The next step is used to make the satellite data comparable with the *in situ* (LST) measurements. In this step, we used (2) for conversion from radiance to LST value:

(2) $$\begin{array}{c} T_{b}=\frac{K_{2}}{\ln\left(K_{1}/L_{\lambda }\right)+1}, \end{array}$$

where

- *K*_1_ = Calibration constant (607.76),
- *K*_2_ = Calibration constant (1260.56),
- *T*_*b*_ = Surface Temperature
- *T*_*b*_ = *T*_*b*_–273 (Conversion of Kelvin to Celsius).

#### 2.4.2. NDVI

NDVI is a simple numerical indicator that can be used for analysing remote sensing measurements, typically but not necessarily from a space platform, and assess whether the target being observed contains live green vegetation or not. The NDVI is calculated as a ratio between measured reflectivity in the red and near infrared portions of the electromagnetic spectrum. These two spectral bands are chosen because they are most affected by the absorption of chlorophyll in leafy green vegetation and by the density of green vegetation on the surface. Also, in red and near-infrared bands, the contrast between vegetation and soil is at a maximum [22]. The NDVI transformation is computed as the ratio of the measured intensities in the red (*R*) and near infrared (NIR) spectral bands (3) using the following formula:

(3) $$\begin{array}{c} \text{NDVI}=\frac{\text{NIR}-\text{red}}{\text{NIR}+\text{red}}. \end{array}$$

#### 2.4.3. VCI

VCI quantifies the weather component. The weather-related NDVI envelope is linearly scaled to 0 for minimum NDVI and 100 for the maximum for each grid cell and week. It is defined as in

(4) $$\begin{array}{c} \text{VCI}=\frac{\text{NDVI}-{\text{NDVI}}_{\min}}{{\text{NDVI}}_{\max}-{\text{NDVI}}_{\min}}, \end{array}$$

where NDVI, NDVI_max⁡_, and NDVI_min⁡_ are the smoothed weekly NDVI, multiyear maximum NDVI and multiyear minimum NDVI, respectively, for each grid cell. VCI changes from 0 to 100, corresponding to changes in vegetation condition from-to extremely unfavourable to optimal. This technique has been improved by converting NDVI with radiation measured in one of the thermal channels and converting brightness temperature into the VCI. This index is being used for estimation of vegetation health and monitoring drought [15].

#### 2.4.4. TVX

TVX combines surface temperature and a normalized difference vegetation index and can be described as follows:

(5) $$\begin{array}{c} \text{TVX}=\frac{\text{LST}}{\text{NDVI}}. \end{array}$$

The combination of NDVI and LST has proved to provide better understanding of drought events with their close inter-relations with surface drought status. The ratio of NDVI and LST, also called TVX, has been proven to be significantly correlated with crop moisture and soil moisture in most climatic and land cover conditions. Results indicated that the use of TVX was a rapid and effective indicator for drought assessment at country or province level [23].

## 3. Results and Discussion

### 3.1. Evaluation of *In Situ* Measurements

**Figure 4:**
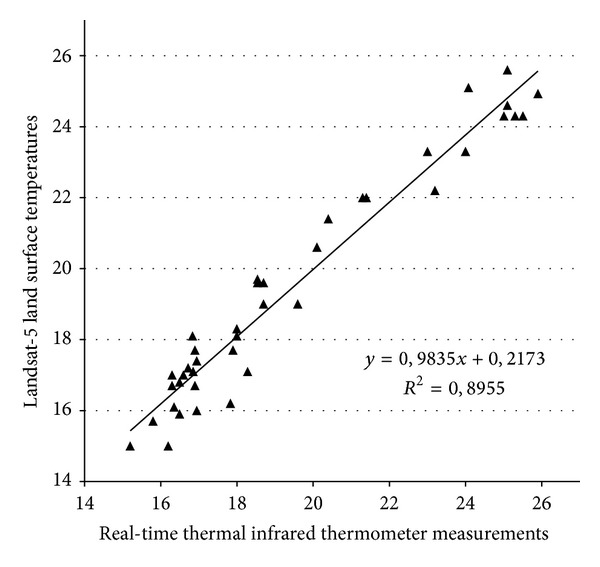
Relationships between real-time (*in situ*) thermal infrared thermometer measurements and land surface temperatures calculated from Landsat-5 thermal infrared band (real-time measurements were collected on August 21, 2011 07:30–11:00 AM, GMT+2). Landsat-5 overpass: 08:21 AM over the Salt Lake, Turkey (path/row = 177/033).

Correlation analysis was performed to examine the relationship between real-time ground and satellite data in the study area using infrared thermometer measurements and thermal infrared band of the Landsat-5 TM image of August 21, 2011. DNs obtained on TM image were first converted to radiance (1) and then to LST values (2) to compare satellite data with real-time *in situ* measurements (Table 2).  Figure 4 summarizes the results of overall conversion process given in Table 2 in detail. Temperature measurements were taken at 40 sample points. Four different types of land cover were identified around the lake: (i) crusted saline soil, (ii) salt, (iii) bare saline soil, and (iv) fallow area (Figure 2). Each measurement resulted from the averaging of about 10 spectra. Spurious spectra were eliminated from data sets during the averaging process.

The relationships between surface (*in situ*) measurements and converted Landsat-5 TIR data are shown in Figure 4. The regression results show that measured surface temperatures and converted Landsat-5 TIR data are in good agreement with *R* ^2^ values about 0.90 in the selected study area (Figure 4). Here, it can be indicated that the use of real-time data set collected on the same day and hours increased consistency between ground and satellite data.

The correlation analysis mentioned above was performed using mean values of infrared thermometer measurements. In the study, we also tested the use of maximum and minimum values temperature measurements for correlation analysis (Table 2). It is experienced that there is no significant difference in the case of use of minimum or maximum values of temperature measurements. Maximum values negligibly increased the correlation, whereas the minimum values decreased the correlation at the same rate for all spectral ranges (i.e., *R*_mean_^2^: 0.8955; *R*_min⁡_^2^: 0.8933; *R*_max⁡_^2^: 0.8983).

### 3.2. Interpretation of Surface Temperature Changes

**Figure 5:**
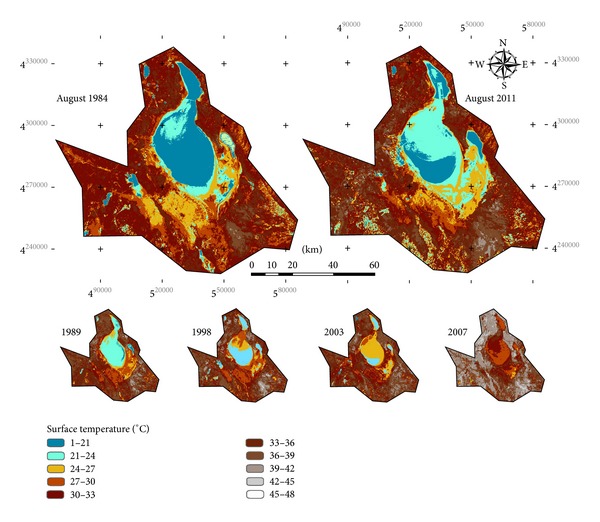
Interpretation of LST changes over Salt Lake Basin Area.

The main outcome of this research has been the production of maps of land surface temperatures for the area of investigation.  Figure 5 shows the spatial and temporal variations in LST in the Salt Lake Basin Area (Turkey). The derived LST values reveal surface temperatures ranging 20–45°C.

**Figure 6:**
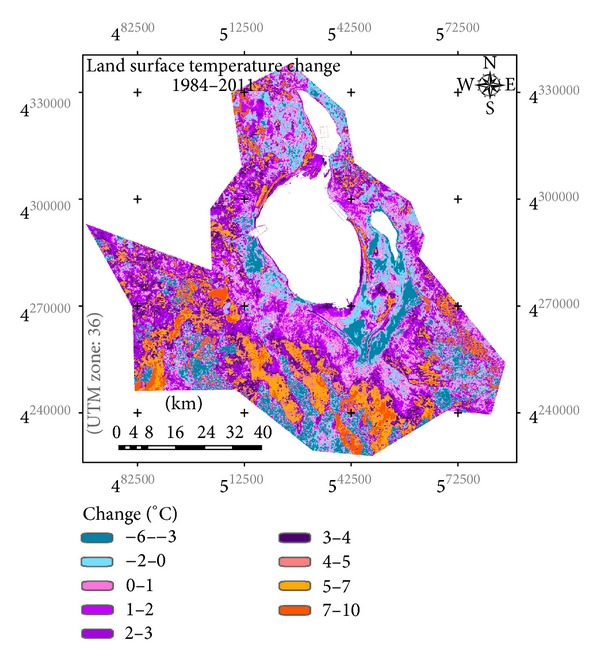
Presentation of LST difference for a 27-year period over Salt Lake Basin Area.

On the basis of the results derived, it is seen that land surface temperature of the study area showed a significant increase over the basin between 1984 and 2011 (Figures 5 and 6). A gradual increase can easily be identified from 1984 to 2007. 2007, in fact, was the year with the highest land surface temperatures over the basin. In 2011, some cooling occurs compared to 2007, but the land surface temperatures were still higher than those of 1984.  Figure 6 presents LST differences for a 28-year period over the Salt Lake Basin Area. This image showing thermal change was derived by image differencing technique. Based on Figure 6, the increases in land surface temperatures were about 2°C from 1984 to 2011.

It is observed that the vegetation and water body areas which act as heat sink have relatively lower temperatures. The densification of the vegetation lowers the temperature as it enhances the evapotranspiration that maintains the heat flux [21]. This demonstrates that agricultural fields where groundwater is used as water supply in summer can easily be monitored using thermal infrared data. For example, agricultural fields located at the south of Salt Lake can be identified in 2011 image. The vegetation and water body in the southeast of the lake had lower temperature in 2011 and Salt Lake, which was partially dried, showed higher temperatures.

It is seen that the land use change is one of the most important factors on the temperature regime of the area and the density of the land use can affect the temperature. The temperature change values are negative in the some parts of the south of the Salt Lake in which areas are densely covered by the agricultural fields. In these areas, the cultivation has increased after the 2000s. The agricultural development decreases the maximum temperature. As parallel to this, current fallow areas for that time of the year show the highest thermal characteristics.

**Figure 7:**
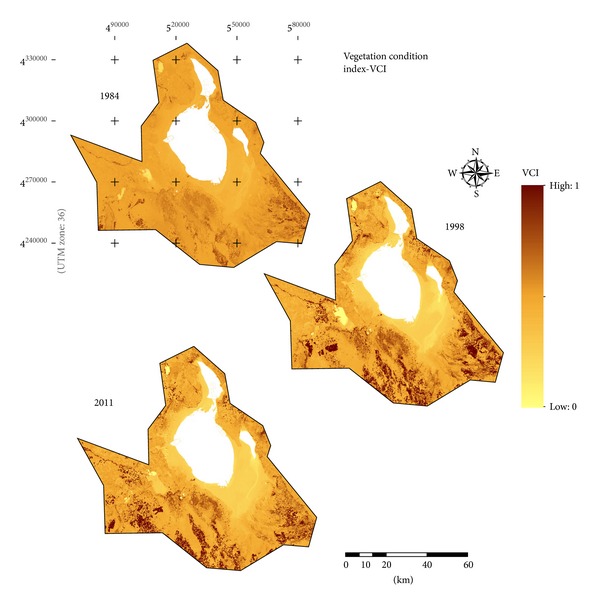
Multitemporal VCI indices maps for the basin.

**Figure 8:**
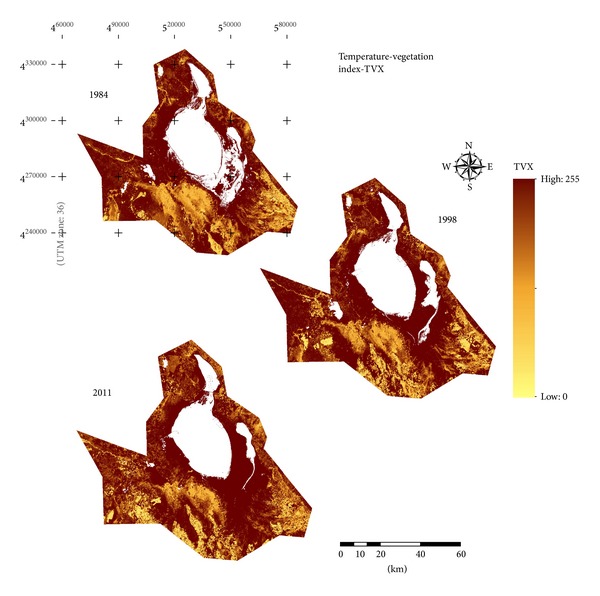
Multitemporal TVX index products for the basin.

From 1984 to 2011, many bare grounds in the south and southeast of the basin were converted to cultivated land.  Figure 7 (VCI) and Figure 8 (TVX) give greatly useful information about the increase in the agricultural fields on the resulting mutlitemporal indices maps. The change paths of cultivated land in the TVX are showed in Figure 8. In 1984, bare grounds were in the lower-right corner of TVX space, corresponding to low vegetation coverage and high temperature. With the development of cultivated land, bare grounds were converted to agricultural fields. From 1984 to 2011, the changes of LST and vegetation cover were obvious. Compared to bare grounds, cultivated lands showed a notable decrease in surface temperatures.

### 3.3. Interpretation of Air Temperature and Precipitation Data

The severity of drought conditions in the Salt Lake Basin Area during the 1984–2011 period was also analysed using climatic data collected in or near the basin. With this approach, we aimed to validate the results obtained using the remotely sensed data. The data used in the analysis included air temperature and precipitation data from three stations (namely, Cihanbeyli, Aksaray, and Kulu stations) (Figure 1). Cihanbeyli is the only weather station within the basin. It is located to the west of Salt Lake. Aksaray and Kulu stations are located to the northwest and southeast of the lake. Data were analysed at annual and monthly (August) timescales. Mann-Kendall trend test [24, 25] and Sen's slope [26] estimation were applied to determine the presence and magnitude of trends in climatic data.

**Figure 9:**
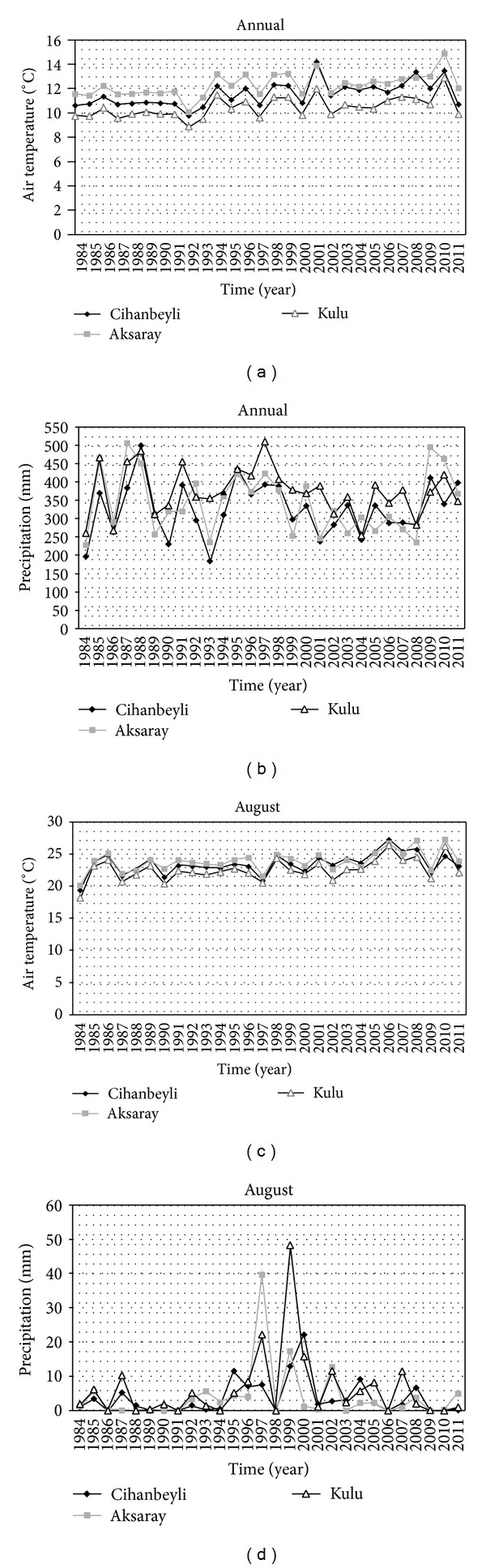
Climatic data analysis for the Salt Lake Basin Area. Air temperature and precipitation data were demonstrated at annual (a and b) and monthly (August) (c and d) timescales.

As can be seen from Figure 9, air temperatures at three stations showed almost consistent patterns both at the annual and monthly timescales. Annual average air temperatures were minimum at all stations in 1992 and maximum in 2010 at Aksaray and Kulu stations and in 1992 at Cihanbeyli station. At Cihanbeyli station, the second highest annual average air temperatures were detected in 2010 and similarly 1992 was the year having the second highest annual average air temperatures at other two stations during the 1984–2011 period. Although it is not possible to establish a direct link between air temperatures and land surface temperatures due to complexity of the processes affecting land surface temperatures, an analysis of air temperatures can provide information about the changes in general climatic conditions in the basin. For the years included in the analysis (i.e., 1984, 1989, 1998, 2003, 2007, and 2011), air temperatures were the lowest in 1984 and the highest in 1998. Air temperatures for August were the lowest in 1984 and the highest in 2007. The trend analysis of annual average air temperature data showed that air temperatures increased in the basin from 1984 to 2011 at a rate of 0.05°C/yr to 0.07°C/yr (corresponding to 1.40°C to 1.96°C for the 28-yr period). The air temperatures in August also showed upward trends (at a rate of 0.09°C/yr or 2.5°C for the 28-yr period at all three stations) during the same period. Trends in annual and August air temperatures were statistically significant at the 0.05 level. Based on this information, we can say that the changes detected in air temperature data at three stations were similar to the range of changes detected with LST data over the basin. The highest air temperatures observed in LST values over the basin were most probably related to August 2007 being much hotter than that of other years.

Precipitation in the basin is directly linked with drought conditions. For the years included in the analysis (i.e., 1984, 1989, 1998, 2003, 2007, and 2011), precipitation was the lowest in 1984 at three stations and the highest in 1998 in Aksaray and Kulu and in 2011 in Cihanbeyli. August, precipitation was generally very low (about 0 to 2 mm/month) at all three stations except for a few years with higher precipitation values. The analysis of precipitation data for the 1984–2011 period showed that precipitation went up at Cihanbeyli station and down at Aksaray and Kulu stations. The rates of changes were low, in the range of −1.83 mm/yr to 0.55 mm/yr (corresponding to −51.2 mm to 15.4 mm for the 28-yr period), and none of the changes in precipitation data were statistically significant.

To summarize, climatic conditions in the Salt Lake Basin Area showed some changes during the 1984–2011 period. It is evident that air temperatures went up in the basin. In terms of precipitation, we do not have that clear picture. The results obtained with climatic data support the findings obtained from the analysis of satellite images. Climatic changes together with land-use changes provide a stronger explanation for LST changes seen in the basin area.

## 4. Conclusions

The improved availability of satellite data having high temporal and spatial resolutions offers many opportunities. Thermal infrared images correlated with real-time ground temperature measurements allow the spatial distribution of LST to be modelled and estimated for an area of interest.

This study examined the relationship between thermal infrared band of the Landsat-5 TM and real-time ground data collected using infrared thermometer. The regression results showed that measured surface temperatures and converted Landsat-5 TIR data were in good agreement with *R* ^2^ values about 0.90 in the selected study area.

According to the results of this investigation, large amount of land has been affected in the basin by especially agricultural facilities due to increasing drought effects and uncontrolled use of ground water in the Salt Lake Basin Area (Turkey). The outcome of this study shows that dramatic land surface temperature changes occurred (about 2°C) in the Salt Lake Basin Area during the 28-year period (1984–2011) along with the increase in agricultural fields. The analysis of climatic data shows that the changes detected in air temperature data in the basin also support these findings. It is evident that air temperatures went up in the basin at a rate of about 1.5–2°C during the same period. Air temperature changes and land-use changes together can be responsible for LST changes seen in the basin.

## Figures and Tables

**Table 1:** Specifications of Landsat data used in the study.

Sensor	Date	Path/row	Band (µm)	Spatial resolution (m)	Number of GCP	RMS error (pixel)
Landsat-5 TM	21/08/2011*	177/033	1: 0.45–0.52	30	43	0.48
	10/08/2007		2: 0.52–0.60		49	0.47
	15/08/2003		3: 0.63–0.69		53	0.45
	01/08/1998		4: 0.76–0.90		50	0.49
	16/08/1989		5: 1.55–1.75		45	0.44
	26/08/1984		7: 2.08–2.35		46	0.43
			6: 10.1–12.5	120		

*The date for the real-time field measurements.

**Table 2:** Real-time infrared thermometer (*in situ*) measurements collected on August 21, 2011 and converted Landsat-5 thermal infrared data.

No.	UTM coordinates (WGS84)		*In situ* measurements (°C)			Landsat-5 TIR data (°C)
	*Y* (m)	*X* (m)	Min	Max	Mean	
1	4313221	531105	24.5	26.8	25.5	24.3
2	4313108	533399	19.8	26.8	24.1	25.1
3	4312886	534540	16.2	17.5	16.9	17.4
4	4313520	534509	16.2	17.5	16.9	16.0
5	4313742	532416	16.3	17.5	16.9	16.7
6	4313964	531814	15.5	17.2	16.4	16.1
7	4313964	530292	15.2	17.0	16.5	15.9
8	4313710	529532	17.0	19.0	18.3	17.1
9	4312918	528993	16.5	17.3	16.8	18.1
10	4311935	528771	15.1	17.5	16.7	17.2
11	4311364	528073	16.3	18.2	17.8	16.2
12	4311935	527851	16.6	19.3	18.5	19.6
13	4312410	528010	15.2	17.2	16.9	17.1
14	4313044	528264	14.8	17.1	16.6	17.0
15	4313774	528137	16.6	18.8	18.0	18.3
16	4313457	527154	20.9	24.8	23.2	22.2
17	4311681	527027	15.1	16.7	16.2	15.0
18	4310572	526710	19.4	22.6	21.3	22.0
19	4309399	526647	21.6	25.7	24.0	23.3
20	4309652	527566	22.5	27.0	25.1	24.6
21	4309050	527154	18.0	20.6	19.6	19.0
22	4308670	526615	18.6	21.5	20.4	21.4
23	4308575	525728	15.2	16.8	16.3	17.0
24	4309240	525664	16.5	18.6	17.9	17.7
25	4310286	525728	14.8	16.2	15.8	15.7
26	4310128	526805	16.6	18.8	18.0	18.1
27	4310128	527091	14.3	15.5	15.2	15.0
28	4310445	527217	17.2	19.6	18.7	19.6
29	4312157	527376	18.4	21.2	20.1	20.6
30	4312791	527788	19.5	22.7	21.4	22.0
31	4313298	527883	22.7	27.3	25.3	24.3
32	4313678	528485	17.1	19.4	18.5	19.7
33	4313964	528803	22.5	27.0	25.1	25.6
34	4314693	529849	15.4	17.0	16.5	16.8
35	4315390	530990	17.2	19.6	18.7	19.0
36	4316183	531370	20.8	24.6	23.0	23.3
37	4316215	532004	15.7	17.5	16.9	17.7
38	4314598	531339	15.2	16.8	16.3	16.7
39	4314027	530926	22.5	26.9	25.0	24.3
40	4313171	530292	23.2	28.0	25.9	24.9

## References

[1] Tran H, Uchihama D, Ochi S, Yasuoka Y (2006). Assessment with satellite data of the urban heat island effects in Asian mega cities. *International Journal of Applied Earth Observation and Geoinformation*.

[2] Carnahan WH, Larson RC (1990). An analysis of an urban heat sink. *Remote Sensing of Environment*.

[3] Carlson TN, Augustine JA, Boland FE (1977). Potential application of satellite temperature measurements in the analysis of land use over urban areas. *Bulletin of the American Meteorological Society*.

[4] Mohan M (2000). Climate change: evaluation of ecological restoration of delhi ridge using remote sensing and GIS technologies. *International Archives of Photogrammetry and Remote Sensing*.

[5] Arnold CL, Gibbons CJ (1996). Impervious surface coverage: the emergence of a key environmental indicator. *Journal of the American Planning Association*.

[6] Ji M, Jensen JR (1999). Effectiveness of subpixel analysis in detecting and quantifying urban imperviousness from landsat thematic mapper imagery. *Geocarto International*.

[7] Ward D, Phinn SR, Murray AT (2000). Monitoring growth in rapidly urbanizing areas using remotely sensed data. *Professional Geographer*.

[8] Voogt JA, Oke TR (2003). Thermal remote sensing of urban climates. *Remote Sensing of Environment*.

[9] Gupta RK, Prasad S, Sai MVRS, Viswanadham TS (1997). The estimation of surface temperature over an agricultural area in the state of Haryana and Panjab, India, and its relationship with the Normalized Difference Vegetation Index (NDVI), using NOAA-AVHRR data. *International Journal of Remote Sensing*.

[10] Falahatkar S, Hosseini SM, Soffianian AR (2011). The relationship between land cover changes and spatial-temporal dynamics of land surface temperature. *Indian Journal of Science and Technology*.

[11] Rajasekar U, Weng Q (2009). Spatio-temporal modelling and analysis of urban heat islands by using Landsat TM and ETM+ imagery. *International Journal of Remote Sensing*.

[12] Muthumanickam D, Kannan P, Kumaraperumal R, Natarajan S, Sivasamy R, Poongodi C (2011). Drought assessment and monitoring through remote sensing and GIS in western tracts of Tamil Nadu, India. *International Journal of Remote Sensing*.

[13] Ghulam A, Qin Q, Kusky T, Li Z-L (2008). A re-examination of perpendicular drought indices. *International Journal of Remote Sensing*.

[14] Lambın EF, Ehrlich D (1995). Combining vegetation indices and surface temperature for land-cover mapping at broad spatial scales. *International Journal of Remote Sensing*.

[15] Singh RP, Roy S, Kogan F (2003). Vegetation and temperature condition indices from NOAA AVHRR data for drought monitoring over India. *International Journal of Remote Sensing*.

[16] Coll C, Caselles V, Galve JM (2005). Ground measurements for the validation of land surface temperatures derived from AATSR and MODIS data. *Remote Sensing of Environment*.

[17] Srivastava PK, Majumdar TJ, Bhattacharya AK (2009). Surface temperature estimation in Singhbhum Shear Zone of India using Landsat-7 ETM+ thermal infrared data. *Advances in Space Research*.

[18] Dymond JR, Shepherd JD (2004). The spatial distribution of indigenous forest and its composition in the Wellington region, New Zealand, from ETM+ satellite imagery. *Remote Sensing of Environment*.

[19] Yamaguchi Y, Naito C (2003). Spectrail indices for lithologic discrimination and mapping by using the ASTER SWIR bands. *International Journal of Remote Sensing*.

[20] Hellweger FL, Schlosser P, Lall U, Weissel JK (2004). Use of satellite imagery for water quality studies in New York Harbor. *Estuarine, Coastal and Shelf Science*.

[21] Joshi JP, Bhatt B (2012). Estimating temporal land surface temperature using remote sensing: a study of vadodara urban, Gujarat. *International Journal of Geology, Earth and Environmental Sciences*.

[22] Cai G, Du M, Liu Y Regional drought monitoring and analyzing using MODIS data—a case study in Yunnan Province.

[23] McVicar TR, Bierwirth PN (2001). Rapidly assessing the 1997 drought in Papua New Guinea using composite AVHRR imagery. *International Journal of Remote Sensing*.

[24] Mann H (1945). Non-parametric tests against trend. *Econometrica*.

[25] Kendall MG (1975). *Rank Correlation Methods*.

[26] Sen P (1968). Estimates of the regression coefficient based on Kendall’s tau. *Journal of American Statistical Association*.

